# (Methanol-κ*O*)(2-methyl-3,5-dinitro­benzoato-κ*O*)triphenyl­tin(IV)

**DOI:** 10.1107/S1600536811011184

**Published:** 2011-04-07

**Authors:** Muhammad Danish, Sabiha Ghafoor, Nazir Ahmad, Wojciech Starosta, Janusz Leciejewicz

**Affiliations:** a Department of Chemistry, University of Gujrat, Hafiz Hayat Campus, Gujrat, 50700 Pakistan; b Department of Chemistry, University of Sargodha, Sargodha 40100, Pakistan; c Institute of Nuclear Chemistry and Technology, ul. Dorodna 16, 03-195 Warszawa, Poland

## Abstract

In the title complex, [Sn(C_6_H_5_)_3_(C_8_H_5_N_2_O_6_)(CH_3_OH)], the Sn(IV) ion is coordinated in a slightly distorted trigonal–bipyramidal geometry by three phenyl ligands in the equatorial plane and by a 2-methyl-3,5-dinitro­benzene­carboxyl­ato ligand and a methanol ligand at the apical sites. In the crystal, complex mol­ecules are linked *via* inter­molecular O—H⋯O hydrogen bonds, forming chains along [100].

## Related literature

For the crystal structures of two triphenyl­tin complexes with a 2,3-dinitro­benzoate ligand, see: Azir-ur-Rehman *et al.* (2006 ▶); Win *et al.* (2006 ▶). For the structure of a tin complex with a 2-methyl­benzoate ligand, see: Danish *et al.* (2010) ▶. For applications of organotin compounds, see: Reisi *et al.* (2006 ▶).
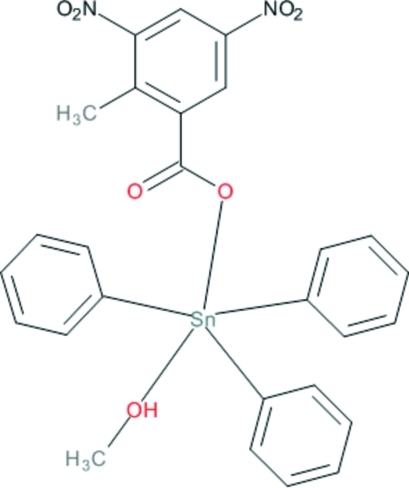


## Experimental

### 

#### Crystal data

[Sn(C_6_H_5_)_3_(C_8_H_5_N_2_O_6_)(CH_4_O)]
*M* *_r_* = 607.17
Monoclinic, 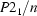

*a* = 8.0597 (16) Å
*b* = 20.094 (4) Å
*c* = 16.022 (3) Å
β = 95.29 (3)°
*V* = 2583.8 (9) Å^3^
*Z* = 4
Mo *K*α radiation
μ = 1.04 mm^−1^
*T* = 293 K
0.31 × 0.23 × 0.07 mm

#### Data collection

Kuma KM4 four-circle diffractometer
Absorption correction: analytical (*CrysAlis RED*; Oxford Diffraction, 2008) ▶ *T*_min_ = 0.842, *T*_max_ = 0.930
6387 measured reflections
6141 independent reflections
3440 reflections with *I* > 2σ(*I*)
*R*_int_ = 0.024
3 standard reflections every 200 reflections  intensity decay: 7.5%

#### Refinement

*R*[*F* ^2^ > 2σ(*F* ^2^)] = 0.037
*wR*(*F* ^2^) = 0.133
*S* = 1.04
6141 reflections
340 parameters
1 restraint
H atoms treated by a mixture of independent and constrained refinement
Δρ_max_ = 0.96 e Å^−3^
Δρ_min_ = −0.78 e Å^−3^

### 

Data collection: *KM-4 Software* (Kuma, 1996 ▶); cell refinement: *KM-4 Software*; data reduction: *DATAPROC* (Kuma, 2001 ▶); program(s) used to solve structure: *SHELXS97* (Sheldrick, 2008 ▶); program(s) used to refine structure: *SHELXL97* (Sheldrick, 2008 ▶); molecular graphics: *SHELXTL* (Sheldrick, 2008 ▶); software used to prepare material for publication: *SHELXTL*.

## Supplementary Material

Crystal structure: contains datablocks I, global. DOI: 10.1107/S1600536811011184/lh5223sup1.cif

Structure factors: contains datablocks I. DOI: 10.1107/S1600536811011184/lh5223Isup2.hkl

Additional supplementary materials:  crystallographic information; 3D view; checkCIF report

## Figures and Tables

**Table 1:** Hydrogen-bond geometry (Å, °)

*D*—H⋯*A*	*D*—H	H⋯*A*	*D*⋯*A*	*D*—H⋯*A*
O51—H51⋯O12^i^	0.81 (2)	1.91 (4)	2.654 (6)	153 (8)

Symmetry code: (i) 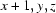.

**Fig. 1.**
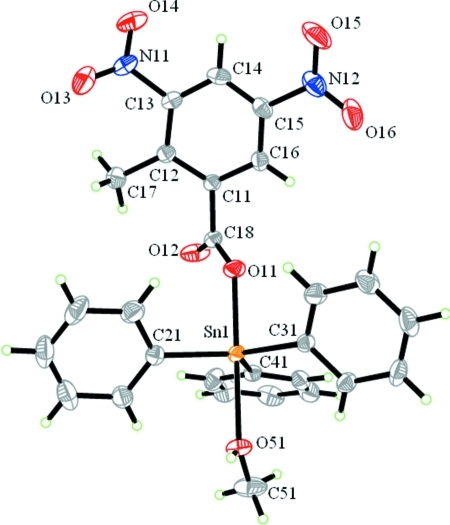
The molecular structure of the title complex shown with 50% probability displacement ellipsoids. For clarity, only bonding benzene ring C atoms are labelled.

**Fig. 2.**
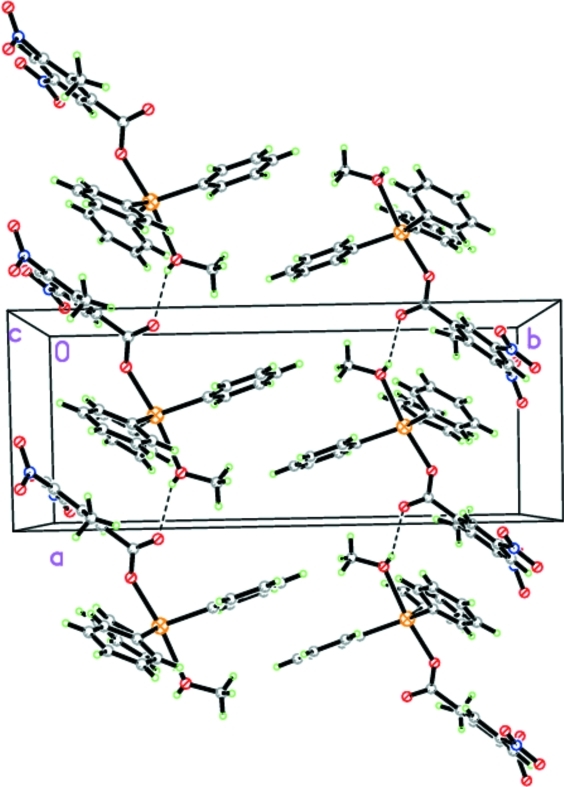
Part of the crystal structure with hydrogen bonds shown as dashed lines.

## supplementary crystallographic information

### Comment

Triphenyltin hydroxide, chloride and acetate compounds are used against a number of fungal diseases to control the attacks of fungi on potato, sugar beat, onion and rice. They are used to protect against tropical diseases in coffee, cocoa and as antifouling agents in paint coating in ships (Reisi *et al.* 2006). The title compound is a continuation of our research invovling the synthesis of new organotin compounds of potential biological activity (Danish *et al.*, 2010).

The molecular structure of the title compound is shown in Fig.1. The structure contains discrete molecules. The central Sn atom is coordinated by three phenyl ligands, a dinitrotoluene carboxylate ligand and a methanol ligand. Three phenyl ligands coordinate the Sn atom through Sn—C bonds whose lengths fall in the narrow range of 2.122((5)Å to 2.132 (5) Å, which was earlier observed in triphenyl tin complexes (Azir-ur-Rehman *et al.*, 2006; Win *et al.*, 2006). The coordinating C atoms form an equatorial plane of a trigonal bipyramid. The Sn atom deviates from this plane by 0.0666 (2) Å. The benzene rings as expected are essentially planar with r.m.s. deviations of 0.0026 (1), 0.0100 (1) and 0.0070 (1)Å and form dihedral angles of 49.8 (2), 57.6 (2) and 5.5 (1)° with the equatorial plane. The dinitrotoluene carboxylate ligand chelates the Sn atom using a single carboxylato O atom, which forms one apex of the coordination bipyramid. The second carboxylato O atom is not coordinated. The Sn—O bond length is 2.162 (3) Å, which is characteristic (Danish *et al.*, 2010). The toluene group is essentially planar [r.m.s. 0.0154 (2) Å] and forms dihedral angles of 40.9 (2) and 5.8 (1)° with nitro groups, N11/O13/O14 and N12/O15/O16 respectively. The dihedral angle between the carboxylate C17/O11/O12 group and the toluene ring is 52.3 (2)°. The methanol hydroxy O51 atom is the apical site with with an Sn—O bond of 2.393 (3) Å. The methanol ligand originates from the solvent used in the course of chemical synthesis. In the crystal, molecules are linked by hydrogen bonds in which the methanol hydroxyl group act as donors and the unccordinated carboxylato O12 atoms acts as an acceptor to form chains propagating along the *a* axis (Fig. 2).

### Experimental

0.124 g.(0.0005 mol) of sodium 3,5-dinitro-toluate and 0.192 g.(0.0005 mol) of triphenyltin chloride were suspended in dry methanol and refluxed for six hours. Sodium chloride, which was formed, was removed by filtration. The solid obtained from the concentration of the filtrate was repeatedly crystallized from chloroform until pale yellow single-crystal plates were found. *M*.p = 369 K. Yield 80%.

### Refinement

The hydroxy group hydrogen atom was located in a difference map and was refined independently with an isotropic displacement parameter. H atoms boned to C atoms were placed in calculated positions with C—H = 0.93 and 0.96Å  and treated as riding on the parent atoms with *U*_iso_(H)= 1.2*U*_eq_(C) or *U*_iso_(H)=1.5*U*_eq_(C_methyl_).

### Crystal data

**Table:**

[Sn(C_6_H_5_)_3_(C_8_H_5_N_2_O_6_)(CH_4_O)]	*F*(000) = 1224
*M**_r_* = 607.17	*D*_x_ = 1.561 Mg m^−^^3^
Monoclinic, *P*2_1_/*n*	Mo *K*α radiation, λ = 0.71073 Å
Hall symbol: -P 2yn	Cell parameters from 25 reflections
*a* = 8.0597 (16) Å	θ = 6–15°
*b* = 20.094 (4) Å	µ = 1.04 mm^−^^1^
*c* = 16.022 (3) Å	*T* = 293 K
β = 95.29 (3)°	Plates, pale yellow
*V* = 2583.8 (9) Å^3^	0.31 × 0.23 × 0.07 mm
*Z* = 4	

### Data collection

**Table:**

Kuma KM4 four-circle diffractometer	3440 reflections with *I* > 2σ(*I*)
Radiation source: fine-focus sealed tube	*R*_int_ = 0.024
graphite	θ_max_ = 28.5°, θ_min_ = 1.6°
profile data from ω/2θ scans	*h* = −10→10
Absorption correction: analytical (*CrysAlis RED*; Oxford Diffraction, 2008)	*k* = 0→26
*T*_min_ = 0.842, *T*_max_ = 0.930	*l* = −20→0
6387 measured reflections	3 standard reflections every 200 reflections
6141 independent reflections	intensity decay: 7.5%

### Refinement

**Table:**

Refinement on *F*^2^	Primary atom site location: structure-invariant direct methods
Least-squares matrix: full	Secondary atom site location: difference Fourier map
*R*[*F*^2^ > 2σ(*F*^2^)] = 0.037	Hydrogen site location: inferred from neighbouring sites
*wR*(*F*^2^) = 0.133	H atoms treated by a mixture of independent and constrained refinement
*S* = 1.04	*w* = 1/[σ^2^(*F*_o_^2^) + (0.0654*P*)^2^ + 3.2267*P*] where *P* = (*F*_o_^2^ + 2*F*_c_^2^)/3
6141 reflections	(Δ/σ)_max_ < 0.001
340 parameters	Δρ_max_ = 0.96 e Å^−^^3^
1 restraint	Δρ_min_ = −0.78 e Å^−^^3^

### Special details

**Table:**

Geometry. All esds (except the esd in the dihedral angle between two l.s. planes) are estimated using the full covariance matrix. The cell esds are taken into account individually in the estimation of esds in distances, angles and torsion angles; correlations between esds in cell parameters are only used when they are defined by crystal symmetry. An approximate (isotropic) treatment of cell esds is used for estimating esds involving l.s. planes.
Refinement. Refinement of F^2^ against ALL reflections. The weighted R-factor wR and goodness of fit S are based on F^2^, conventional R-factors R are based on F, with F set to zero for negative F^2^. The threshold expression of F^2^ > 2sigma(F^2^) is used only for calculating R-factors(gt) etc. and is not relevant to the choice of reflections for refinement. R-factors based on F^2^ are statistically about twice as large as those based on F, and R- factors based on ALL data will be even larger.

### Fractional atomic coordinates and isotropic or equivalent isotropic displacement parameters (Å^2^)

**Table:**

	*x*	*y*	*z*	*U*_iso_*/*U*_eq_	
Sn1	0.47331 (3)	0.246055 (14)	0.773898 (18)	0.03309 (10)	
O11	0.2416 (4)	0.30063 (18)	0.7557 (2)	0.0474 (8)	
C36	0.6404 (6)	0.2620 (3)	0.6100 (3)	0.0451 (11)	
H36	0.6976	0.2237	0.6283	0.054*	
C11	−0.0109 (5)	0.3536 (2)	0.7691 (3)	0.0333 (9)	
C12	−0.0853 (6)	0.3849 (2)	0.8344 (3)	0.0373 (10)	
C21	0.5163 (6)	0.2838 (2)	0.8981 (3)	0.0383 (10)	
C31	0.5350 (5)	0.2938 (2)	0.6620 (3)	0.0350 (9)	
C15	−0.1386 (7)	0.4289 (2)	0.6689 (3)	0.0443 (11)	
O12	0.0567 (5)	0.24487 (18)	0.8209 (3)	0.0659 (12)	
N12	−0.1578 (8)	0.4552 (3)	0.5823 (3)	0.0647 (14)	
C41	0.3905 (5)	0.1462 (2)	0.7589 (3)	0.0372 (10)	
C16	−0.0334 (6)	0.3769 (2)	0.6871 (3)	0.0396 (10)	
H16	0.0228	0.3573	0.6454	0.047*	
C42	0.3836 (7)	0.1148 (3)	0.6825 (3)	0.0498 (12)	
H42	0.4173	0.1376	0.6364	0.060*	
C13	−0.1887 (6)	0.4382 (2)	0.8096 (3)	0.0425 (11)	
C18	0.1024 (5)	0.2939 (2)	0.7841 (3)	0.0358 (9)	
C32	0.4572 (7)	0.3511 (3)	0.6343 (4)	0.0520 (13)	
H32	0.3903	0.3736	0.6693	0.062*	
C14	−0.2212 (6)	0.4600 (2)	0.7284 (4)	0.0472 (12)	
H14	−0.2960	0.4944	0.7147	0.057*	
N11	−0.2681 (6)	0.4779 (2)	0.8730 (4)	0.0592 (13)	
C34	0.5750 (8)	0.3438 (4)	0.5043 (4)	0.0663 (18)	
H34	0.5852	0.3600	0.4506	0.080*	
C26	0.4510 (7)	0.3445 (3)	0.9175 (4)	0.0602 (15)	
H26	0.3961	0.3704	0.8756	0.072*	
C25	0.4669 (9)	0.3670 (4)	0.9993 (5)	0.080 (2)	
H25	0.4226	0.4082	1.0117	0.096*	
C22	0.5990 (7)	0.2460 (3)	0.9619 (3)	0.0532 (12)	
H22	0.6448	0.2050	0.9500	0.064*	
C33	0.4755 (8)	0.3764 (3)	0.5555 (4)	0.0615 (16)	
H33	0.4205	0.4152	0.5375	0.074*	
C45	0.2907 (8)	0.0463 (3)	0.8180 (5)	0.0641 (16)	
H45	0.2612	0.0229	0.8645	0.077*	
C35	0.6596 (7)	0.2877 (3)	0.5310 (4)	0.0573 (15)	
H35	0.7300	0.2668	0.4964	0.069*	
C44	0.2799 (8)	0.0158 (3)	0.7412 (5)	0.0673 (18)	
H44	0.2407	−0.0276	0.7356	0.081*	
C46	0.3443 (7)	0.1107 (3)	0.8273 (4)	0.0543 (14)	
H46	0.3498	0.1307	0.8798	0.065*	
O16	−0.0735 (7)	0.4294 (3)	0.5316 (3)	0.0816 (15)	
C17	−0.0513 (7)	0.3627 (3)	0.9229 (3)	0.0569 (14)	
H171	−0.0159	0.3170	0.9241	0.085*	
H172	−0.1509	0.3668	0.9511	0.085*	
H173	0.0349	0.3898	0.9507	0.085*	
C43	0.3267 (8)	0.0492 (3)	0.6729 (4)	0.0649 (17)	
H43	0.3209	0.0286	0.6207	0.078*	
O14	−0.4095 (6)	0.4972 (3)	0.8526 (4)	0.0997 (18)	
O13	−0.1901 (7)	0.4907 (3)	0.9382 (4)	0.0993 (19)	
O15	−0.2542 (8)	0.5001 (3)	0.5664 (3)	0.0948 (18)	
C23	0.6126 (9)	0.2701 (4)	1.0438 (4)	0.073 (2)	
H23	0.6677	0.2451	1.0866	0.088*	
C24	0.5455 (9)	0.3303 (5)	1.0615 (4)	0.084 (3)	
H24	0.5540	0.3460	1.1163	0.101*	
O51	0.7475 (4)	0.19935 (17)	0.7928 (3)	0.0485 (9)	
C51	0.7965 (8)	0.1327 (3)	0.7876 (5)	0.079 (2)	
H51A	0.7885	0.1113	0.8406	0.118*	
H51B	0.9096	0.1307	0.7734	0.118*	
H51C	0.7251	0.1104	0.7452	0.118*	
H51	0.825 (7)	0.223 (3)	0.808 (5)	0.10 (3)*	

### Atomic displacement parameters (Å^2^)

**Table:**

	*U*^11^	*U*^22^	*U*^33^	*U*^12^	*U*^13^	*U*^23^
Sn1	0.03123 (15)	0.03696 (16)	0.03213 (15)	0.00077 (14)	0.00859 (10)	0.00146 (15)
O11	0.0307 (17)	0.060 (2)	0.053 (2)	0.0075 (15)	0.0130 (15)	0.0109 (17)
C36	0.038 (2)	0.055 (3)	0.043 (3)	0.000 (2)	0.009 (2)	−0.003 (2)
C11	0.026 (2)	0.040 (2)	0.035 (2)	0.0011 (17)	0.0071 (17)	0.0017 (19)
C12	0.031 (2)	0.040 (2)	0.041 (3)	−0.0014 (18)	0.0049 (19)	0.000 (2)
C21	0.036 (2)	0.040 (2)	0.040 (3)	−0.0054 (19)	0.0093 (19)	0.002 (2)
C31	0.029 (2)	0.041 (2)	0.036 (2)	−0.0016 (18)	0.0059 (18)	−0.0010 (19)
C15	0.053 (3)	0.036 (2)	0.041 (3)	−0.007 (2)	−0.008 (2)	0.006 (2)
O12	0.046 (2)	0.051 (2)	0.105 (4)	0.0119 (17)	0.028 (2)	0.029 (2)
N12	0.088 (4)	0.049 (3)	0.055 (3)	−0.005 (3)	−0.011 (3)	0.012 (2)
C41	0.030 (2)	0.040 (2)	0.042 (3)	0.0010 (18)	0.0029 (19)	−0.001 (2)
C16	0.038 (2)	0.048 (3)	0.033 (2)	−0.002 (2)	0.004 (2)	0.001 (2)
C42	0.050 (3)	0.054 (3)	0.044 (3)	0.002 (2)	−0.004 (2)	−0.001 (2)
C13	0.037 (3)	0.040 (2)	0.051 (3)	0.0043 (19)	0.007 (2)	−0.005 (2)
C18	0.031 (2)	0.038 (2)	0.039 (2)	0.0019 (17)	0.0076 (18)	−0.0009 (19)
C32	0.047 (3)	0.055 (3)	0.058 (3)	0.004 (2)	0.019 (3)	0.015 (3)
C14	0.045 (3)	0.034 (2)	0.062 (4)	0.006 (2)	−0.005 (2)	0.006 (2)
N11	0.056 (3)	0.051 (3)	0.072 (4)	0.012 (2)	0.016 (3)	−0.012 (2)
C34	0.065 (4)	0.097 (5)	0.038 (3)	−0.014 (4)	0.008 (3)	0.019 (3)
C26	0.054 (3)	0.059 (3)	0.067 (4)	0.006 (3)	0.004 (3)	−0.015 (3)
C25	0.063 (4)	0.096 (5)	0.084 (5)	−0.007 (4)	0.015 (4)	−0.057 (5)
C22	0.050 (3)	0.063 (3)	0.047 (3)	−0.002 (3)	0.004 (2)	0.001 (3)
C33	0.057 (3)	0.074 (4)	0.055 (4)	0.001 (3)	0.008 (3)	0.029 (3)
C45	0.069 (4)	0.048 (3)	0.078 (4)	−0.010 (3)	0.015 (3)	0.012 (3)
C35	0.054 (3)	0.082 (4)	0.039 (3)	−0.012 (3)	0.019 (2)	−0.010 (3)
C44	0.063 (4)	0.037 (3)	0.098 (5)	−0.005 (3)	−0.015 (4)	0.000 (3)
C46	0.061 (3)	0.049 (3)	0.055 (3)	−0.004 (3)	0.021 (3)	0.000 (3)
O16	0.120 (4)	0.082 (3)	0.044 (2)	−0.004 (3)	0.010 (3)	0.013 (2)
C17	0.058 (3)	0.070 (4)	0.043 (3)	0.008 (3)	0.011 (3)	0.006 (3)
C43	0.075 (4)	0.052 (3)	0.063 (4)	0.004 (3)	−0.020 (3)	−0.016 (3)
O14	0.070 (3)	0.119 (4)	0.112 (4)	0.052 (3)	0.019 (3)	−0.014 (4)
O13	0.104 (4)	0.118 (4)	0.076 (4)	0.029 (3)	0.009 (3)	−0.039 (3)
O15	0.135 (5)	0.069 (3)	0.074 (3)	0.019 (3)	−0.026 (3)	0.026 (3)
C23	0.063 (4)	0.113 (6)	0.042 (3)	−0.027 (4)	−0.003 (3)	0.004 (3)
C24	0.060 (4)	0.148 (8)	0.044 (4)	−0.026 (5)	0.008 (3)	−0.033 (4)
O51	0.0348 (19)	0.0401 (18)	0.071 (3)	0.0065 (15)	0.0049 (18)	−0.0007 (18)
C51	0.060 (4)	0.045 (3)	0.129 (7)	0.015 (3)	−0.006 (4)	−0.011 (4)

### Geometric parameters (Å, °)

**Table:**

Sn1—C41	2.121 (5)	C14—H14	0.9300
Sn1—C21	2.128 (5)	N11—O13	1.196 (7)
Sn1—C31	2.132 (5)	N11—O14	1.219 (6)
Sn1—O11	2.162 (3)	C34—C35	1.366 (9)
Sn1—O51	2.394 (3)	C34—C33	1.366 (9)
O11—C18	1.257 (5)	C34—H34	0.9300
C36—C35	1.389 (8)	C26—C25	1.380 (9)
C36—C31	1.397 (6)	C26—H26	0.9300
C36—H36	0.9300	C25—C24	1.351 (11)
C11—C16	1.391 (6)	C25—H25	0.9300
C11—C12	1.400 (6)	C22—C23	1.394 (9)
C11—C18	1.514 (6)	C22—H22	0.9300
C12—C13	1.393 (6)	C33—H33	0.9300
C12—C17	1.489 (7)	C45—C44	1.369 (9)
C21—C26	1.376 (7)	C45—C46	1.367 (8)
C21—C22	1.393 (7)	C45—H45	0.9300
C31—C32	1.365 (7)	C35—H35	0.9300
C15—C16	1.360 (7)	C44—C43	1.367 (10)
C15—C14	1.364 (8)	C44—H44	0.9300
C15—N12	1.479 (7)	C46—H46	0.9300
O12—C18	1.222 (6)	C17—H171	0.9600
N12—O15	1.203 (7)	C17—H172	0.9600
N12—O16	1.221 (7)	C17—H173	0.9600
C41—C42	1.374 (7)	C43—H43	0.9300
C41—C46	1.387 (7)	C23—C24	1.364 (11)
C16—H16	0.9300	C23—H23	0.9300
C42—C43	1.400 (8)	C24—H24	0.9300
C42—H42	0.9300	O51—C51	1.401 (6)
C13—C14	1.374 (7)	O51—H51	0.81 (2)
C13—N11	1.482 (7)	C51—H51A	0.9600
C32—C33	1.382 (7)	C51—H51B	0.9600
C32—H32	0.9300	C51—H51C	0.9600
			
C41—Sn1—C21	117.76 (18)	O13—N11—C13	119.3 (5)
C41—Sn1—C31	115.18 (18)	O14—N11—C13	116.1 (5)
C21—Sn1—C31	126.54 (17)	C35—C34—C33	120.7 (5)
C41—Sn1—O11	101.88 (15)	C35—C34—H34	119.7
C21—Sn1—O11	90.55 (17)	C33—C34—H34	119.7
C31—Sn1—O11	85.63 (15)	C21—C26—C25	120.1 (7)
C41—Sn1—O51	85.44 (15)	C21—C26—H26	120.0
C21—Sn1—O51	87.29 (17)	C25—C26—H26	120.0
C31—Sn1—O51	89.91 (15)	C24—C25—C26	121.2 (7)
O11—Sn1—O51	172.54 (13)	C24—C25—H25	119.4
C18—O11—Sn1	133.3 (3)	C26—C25—H25	119.4
C35—C36—C31	119.9 (5)	C21—C22—C23	119.5 (6)
C35—C36—H36	120.1	C21—C22—H22	120.3
C31—C36—H36	120.1	C23—C22—H22	120.3
C16—C11—C12	121.7 (4)	C34—C33—C32	119.4 (6)
C16—C11—C18	116.1 (4)	C34—C33—H33	120.3
C12—C11—C18	122.1 (4)	C32—C33—H33	120.3
C13—C12—C11	114.8 (4)	C44—C45—C46	120.9 (6)
C13—C12—C17	123.7 (5)	C44—C45—H45	119.5
C11—C12—C17	121.5 (4)	C46—C45—H45	119.5
C26—C21—C22	119.0 (5)	C34—C35—C36	119.9 (6)
C26—C21—Sn1	119.7 (4)	C34—C35—H35	120.0
C22—C21—Sn1	121.2 (4)	C36—C35—H35	120.0
C32—C31—C36	118.6 (5)	C43—C44—C45	120.0 (5)
C32—C31—Sn1	121.1 (4)	C43—C44—H44	120.0
C36—C31—Sn1	119.8 (3)	C45—C44—H44	120.0
C16—C15—C14	122.4 (5)	C45—C46—C41	120.4 (6)
C16—C15—N12	119.0 (5)	C45—C46—H46	119.8
C14—C15—N12	118.5 (5)	C41—C46—H46	119.8
O15—N12—O16	124.5 (6)	C12—C17—H171	109.5
O15—N12—C15	118.4 (6)	C12—C17—H172	109.5
O16—N12—C15	117.1 (5)	H171—C17—H172	109.5
C42—C41—C46	118.4 (5)	C12—C17—H173	109.5
C42—C41—Sn1	121.4 (4)	H171—C17—H173	109.5
C46—C41—Sn1	120.1 (4)	H172—C17—H173	109.5
C15—C16—C11	119.1 (5)	C44—C43—C42	119.1 (6)
C15—C16—H16	120.5	C44—C43—H43	120.4
C11—C16—H16	120.5	C42—C43—H43	120.4
C41—C42—C43	121.1 (6)	C24—C23—C22	120.3 (7)
C41—C42—H42	119.5	C24—C23—H23	119.8
C43—C42—H42	119.5	C22—C23—H23	119.8
C14—C13—C12	124.8 (5)	C25—C24—C23	119.9 (6)
C14—C13—N11	114.9 (5)	C25—C24—H24	120.0
C12—C13—N11	120.2 (5)	C23—C24—H24	120.0
O12—C18—O11	125.5 (4)	C51—O51—Sn1	129.0 (4)
O12—C18—C11	120.7 (4)	C51—O51—H51	112 (6)
O11—C18—C11	113.8 (4)	Sn1—O51—H51	119 (6)
C31—C32—C33	121.5 (5)	O51—C51—H51A	109.5
C31—C32—H32	119.3	O51—C51—H51B	109.5
C33—C32—H32	119.3	H51A—C51—H51B	109.5
C15—C14—C13	117.0 (4)	O51—C51—H51C	109.5
C15—C14—H14	121.5	H51A—C51—H51C	109.5
C13—C14—H14	121.5	H51B—C51—H51C	109.5
O13—N11—O14	124.6 (6)		

### Hydrogen-bond geometry (Å, °)

**Table:**

*D*—H···*A*	*D*—H	H···*A*	*D*···*A*	*D*—H···*A*
O51—H51···O12^i^	0.81 (2)	1.91 (4)	2.654 (6)	153 (8)

Symmetry codes: (i) *x*+1, *y*, *z*.
